# A brain-health framework for PTSD: integrating digital biomarkers into multimodal assessment

**DOI:** 10.1038/s41746-026-02801-4

**Published:** 2026-05-30

**Authors:** Edel McGlanaghy, Dylan Powell, Tony Robertson

**Affiliations:** 1 Adult Psychological Therapies, NHS Forth Valley Mayfield, Falkirk Community Hospital, Falkirk, UK; 2 School of Health & Social Care, Edinburgh Napier University, Sighthill, UK; 3 Faculty of Health Sciences and Sport, University of Stirling, Stirling, UK; 4 General Practice and Primary Care, School of Health & Wellbeing, University of Glasgow, Glasgow, UK

**Keywords:** Engineering, Health care, Neuroscience, Psychology, Psychology

## Abstract

We propose a novel framework and measurement model that frames post-traumatic stress disorder (PTSD) in the context of brain health capital, utilising advances in smart devices (wearables, phones) and AI to better capture the multimodal physiological, behavioural, and cognitive features of PTSD. Patients would be provided with complex information in a meaningful way that could provide a unique opportunity to identify personal change mechanisms and support empowered, patient-led decision-making.

Traumatic exposure is nearly universal, with over 70% of adults worldwide experiencing at least one traumatic event in their lifetime^1^. While most recover, around 5–6% of trauma-exposed individuals will go on to develop Post-Traumatic Stress Disorder (PTSD)^2^, a mental health condition that can arise in the aftermath of stressful, frightening or distressing events. The symptoms of PTSD include re-experiencing traumatic memories (flashbacks, nightmares, etc.), avoidance of people/places and emotional numbing and/or hyperarousal (irritability, angry outbursts etc.). PTSD and Complex PTSD (C-PTSD: PTSD symptoms alongside problems with sense of self, emotional dysregulation and interpersonal difficulties, typically due to chronic or repeated trauma exposures) arise due to interrupted memory processing of the traumatic event, leading to the symptoms noted above. The combination of psychological, physical and social implications can lead to feelings of disempowerment i.e. learned helplessness/mental defeat^3,4^ and can become associated with one’s personal identity as being defective or ‘unable’, thereby interfering with psychological therapy which requires active engagement in recovery. PTSD also has a physiological impact and is associated with a 40–60% higher risk of coronary heart disease^5^, elevated inflammation and metabolic dysfunction^6^, and accounts for a substantial share of years lived with disability in conflict-affected and high-income populations alike^7,8^. PTSD alters brain network connectivity, endocrine and immune responses, and behaviour - affecting every domain of functioning^9–11^. This interconnection positions PTSD not solely as a psychiatric disorder, but as a whole-system disturbance of *brain health*, with implications that extend from individual well-being to population health and economics^12^. We posit that PTSD and C-PTSD are therefore best understood, managed and treated within a wider system of brain health. Below we expand on the causes and consequences of PTSD and how we envisage reframing using a brain health lens, incorporating digital twin technology^13^.

## Risk factors and maintenance factors for PTSD

The majority of risk factor studies for PTSD are based on self-reported characteristics across a range of heterogeneous samples, geographic populations and types of traumatic experiences. And while many cognitive, social, demographic and physical factors have been investigated and links identified, evidence is mixed about their role in the development of, maintenance of, and impact on recovery from, PTSD symptoms. This makes their application to clinical practice impractical and impersonal.

An umbrella review reported only three characteristics that were convincing in predicting PTSD: indigenous status in America, history of chronic disease, and family history of psychiatric disorders^14^. Being female, and experience of more complex traumatic experiences (were also highly suggestive of association with PTSD symptoms. Socioeconomic status, prior abuse and adversity had a weak association.

A more in-depth meta-analysis of pre-trauma risk factors, found that psychopathology had a moderate effect on PTSD symptoms after a subsequent traumatic event^15^. Cognitive factors (such as intelligence, memory), psychophysiology (such as low physical health and low cortisol stress reactivity) and personality traits (such as neuroticism and anxiety) had smaller effects, with a weak effect of environmental factors (e.g. low social support and previous trauma). Coping styles and demographics had no significant association. In the individual factors, sleep complaints, negative emotionality and general distress had the highest associations.

A review of digital factors and PTSD identified that ‘wake after sleep onset’ and relative ‘amplitude of physical activity’ were associated with PTSD symptoms however concluded that further high quality studies of within-person metrics are required, across the current ‘4 dimensions; sleep, location, physical activity and social activity’^13^. Such evidence is required to understand the complex interplay of individual, trauma and social characteristics that can lead to PTSD and/or contribute to recovery. Digital measurement allows for behavioural and ‘brain health’ factors, which provides an opportunity to move beyond demographic descriptors.

Overall, existing evidence suggests that traditional risk factors have limited predictive utility at the individual level and that PTSD arises from complex, interacting biological, psychological, and social processes. This reinforces the need for multimodal, longitudinal approaches capable of capturing dynamic changes over time, as proposed in this framework.

## Treating PTSD

National Institute for Health and Care Excellence (NICE) guidelines in the UK recommend that psychological therapies such as Eye Movement Desensitization and Reprocessing (EMDR) and Cognitive Behavioural Therapy (CBT) are useful first-line treatments for PTSD^16^, both of which target memories of the traumatic event(s) directly. As C-PTSD is a relatively new diagnosis, there is less evidence for treatment, however recently published Scottish recommendations for evidence based psychological therapies, the NHS Education for Scotland (NES) Matrix for C-PTSD indicates that therapies for PTSD are deemed efficacious, alongside skills-focused interventions^17^. Further, these recommendations state that “a biopsychosocial approach to the management of C-PTSD is essential” and that social factors such as safety, risk, social isolation, housing, finances, education, and employment are all important outcomes to be considered^17^. Managing these complex factors and issues requires a wider systems approach, which cannot be addressed within psychological therapies alone and require innovative strategies to manage coherently.

Psychological treatment of PTSD considers all aspects of health and wellbeing, such as sleep, exercise, nutrition and social connection. These elements can be harnessed to support recovery, or where there are deficits, identified as blocks to change. Sleep provides a neat example, and as noted above, is recognised as an important factor in maintaining PTSD. Poor sleep can exacerbate all mental health symptoms and is highly linked with depressive symptoms. Conversely, poor sleep can be caused by anxiety and PTSD specific symptoms such as re-experiencing and nightmares. The resultant exhaustion can compound a sense of failure/lack of motivation, which can further impact brain health and impede the opportunity for ‘natural’ recovery. Detailed exploration of how sleep interacts with PTSD symptoms, treatment and recovery, from a digital twin (a dynamic, data-driven representation of an individual’s state over time, integrating multimodal behavioural, physiological, and contextual data) for example, could illuminate the viability of alternative non-psychological pathways to reduce distress^18^. More detailed information from a such an innovation twin could be highly valuable to the field in understanding the interplay between these variables alongside, and before, therapy is considered, in turn providing potential to improve efficiency and support earlier intervention, although formal economic evaluation is required. Reducing the burden on NHS psychological therapies services would be a welcome outcome of any new innovation in PTSD understanding.

## PTSD and brain health

We posit that PTSD and C-PTSD are best understood within a wider system of brain health. The World Health Organization defines brain health as “the state of brain functioning across cognitive, sensory, social-emotional, behavioural, and motor domains, allowing a person to realise their full potential across the lifecourse”^19^. This makes clear that PTSD does not only affect memory or mood, but also interacts with cognition, physical health, relationships, and social participation, which in turn impacts brain health.

From this perspective, PTSD represents a significant disruption to *brain health capital*, the cognitive, emotional, and functional resources that can be built up, maintained, or depleted across life^12^. Intrusive memories, hyperarousal, and avoidance behaviours reduce cognitive capacity, alter physiological regulation, and limit social and economic opportunities. This whole-system disruption highlights the need to situate trauma care within a broader brain health agenda rather than within narrow psychiatric and psychological therapy silos.

**Fig. 1:**
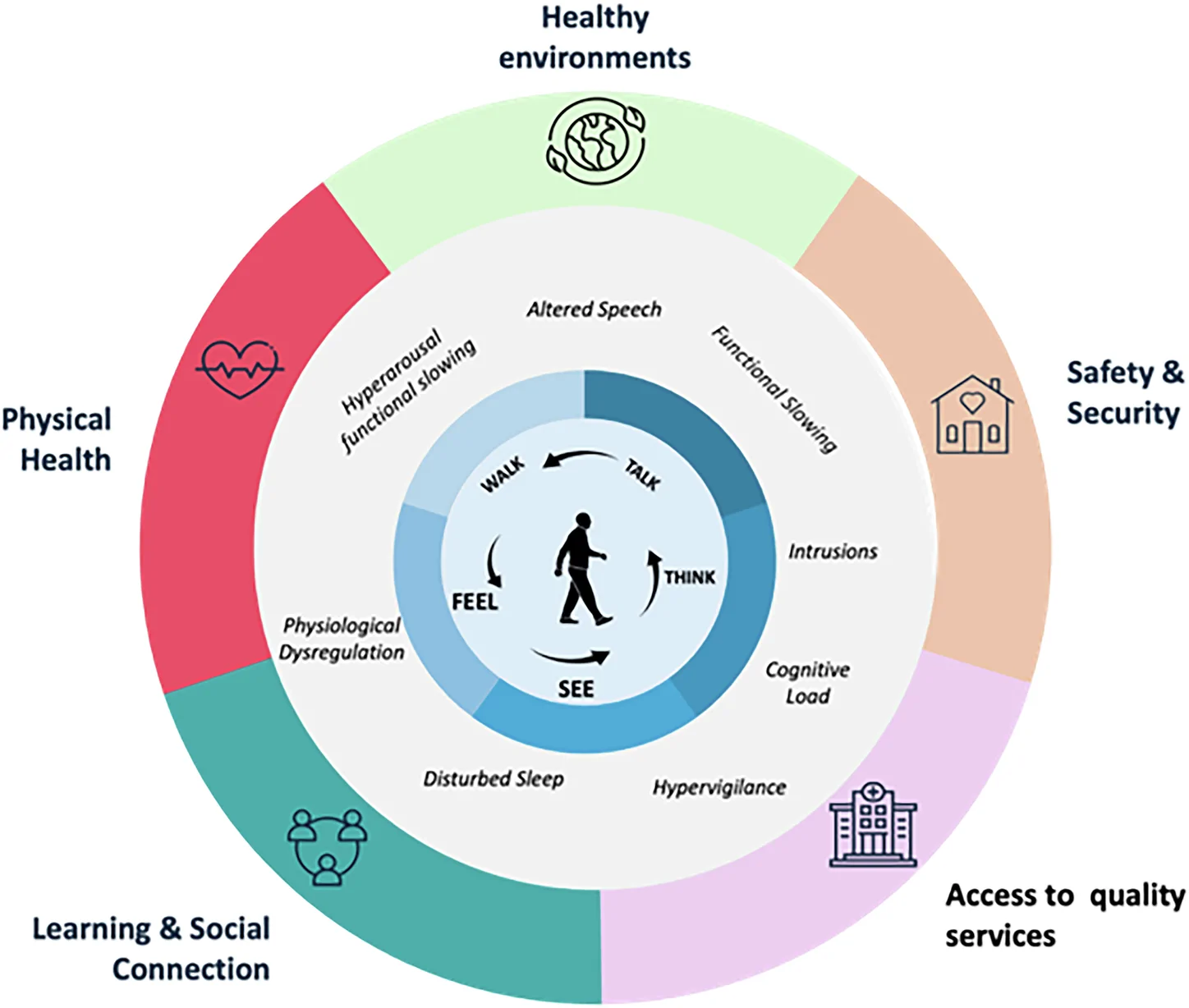
The “Walk, Talk, Think, See, Feel” framework. Parts of this figure utilises images from The Noun Project https://thenounproject.com/, all licensed under CC BY-SA.

Digital biomarkers provide a practical way to capture and monitor this broader framing. The “walk, talk, think, see, feel” framework developed by one of the authors identifies five domains where digital signals can serve as sensitive, real-world markers of brain health (Fig. 1)^20^. Gait performance, including speed and variability, is sensitive to cognitive load, with dual-task conditions demonstrating impairments due to attentional interference. In PTSD, where hyperarousal and attentional disruption are core features, similar alterations in mobility may be observed^21^. Wearables can capture subtle changes in mobility and coordination, offering early indicators of stress-related functional decline (**WALK**). Speech carries acoustic signatures of distress such as altered pitch, prosody, or pauses. Passive voice monitoring could detect heightened arousal or avoidance of trauma-related topics. This could be especially relevant in a therapeutic setting (**TALK**). Cognitive performance, particularly in attention, memory, and executive function, is commonly affected by PTSD. Smartphone-based cognitive tasks can provide ecologically valid measures outside the clinic (**THINK**). Visual attention patterns, including hypervigilance to threat or difficulty disengaging from reminders of trauma, can be tracked using eye-tracking or smartphone-based tests. Sleep-wake monitoring also falls within this domain, as disturbed sleep is one of the most common PTSD symptoms (**SEE**). Self-reported mood, stress, and trauma-related intrusions provide important context. Passive monitoring of physiological signals (heart rate variability, galvanic skin response) offers complementary insights into affective states and allows for biofeedback about therapeutic and daily experiences (**FEEL**).

Many of the signals captured within this framework represent shared phenotypic expressions of disrupted brain health that are not specific to a single diagnostic category. Rather than relying on individual markers, differentiation between conditions such as PTSD, depression, and anxiety is likely to emerge from patterns, temporal dynamics, and contextual associations across domains. In line with emerging efforts to develop formal mental health ontologies, this framework may support the structuring of observable signals and their relationships to underlying mechanisms, enabling more consistent integration and interpretation across studies^22^.

## Translating theory into measurement

Changes in technology continue to provide new opportunities for measuring our biological systems. In recent years these technological advancements have meant being able to access biomarkers using less invasive methods of collection such as through dried blood spots and digital smart devices^23–25^. We can now also measure molecules at scale relatively cheaply and quickly, such as those that span the ‘omics’, providing never-before-seen insights into the complex interplay across our biological systems^26^. Advancements in wearable devices and smartphone apps are broadening the possibilities for tracking the likes of sleep patterns, physical performance and movement, cardiovascular metrics like blood pressure, and various aspects of brain function and development^13,27^.

However, the sheer volume of biomarker data generated by modern techniques requires advanced computational tools for practical analyses. While consumer-grade devices may introduce measurement noise, particularly in domains such as movement and physiological sensing, longitudinal within-person trends and multimodal triangulation may help mitigate these limitations.

Developing a holistic framework for PTSD identification and monitoring faces these same challenges. Given the complexity of PTSD, in terms of its determinants, development, symptoms and timing of onset, multiple levels of measurement across biological, psychological, sociodemographic and behavioural characteristics, as well as over time, are required. These measures would also need to be integrated meaningfully for both practitioners and patients. However, having multiple, independent inputs and outputs, that both take on various forms, generates significant complexity, as each input can influence multiple outputs and vice versa. This requires complex models and specialised techniques to manage these interactions without generating biased results. Integration of multimodal data may be supported by time-series modelling, multimodal machine learning, and latent variable approaches to generate interpretable summaries of brain health domains. Integrating Artificial Intelligence (AI) into our existing suite of tools and methods offers promise in helping us better understand the complex interplay within and between our external, social worlds and our internal, biological systems. In Table 1 we highlight options for measures that can capture each domain of our framework, although this list is not exhaustive and simply aims to highlight the possibilities. PTSD specific symptoms will also be assessed using validated clinical outcome measures, such as PCL-5^28^.

**Table 1:** Converting the “Walk, Talk, Think, See, Feel” framework into a practical measurement tool

Domain	Concept of Interest	Relevant Outcome Measure	Digital Measure / Technology	Clinical Meaning / Application
**Walk**	Hyperarousal; functional impairment	Gait speed; stride variability; step count	Wearables / IMUs (accelerometer, gyroscope)	Detect attentional disruption and motor slowing; monitor rehabilitation and daily activity
**Talk**	Re-experiencing; avoidance; distress expression	Speech fluency, pitch, prosody, pauses	Smartphone/app acoustic analysis; natural language processing	Identify distress or avoidance; track therapy engagement; passive monitoring in daily life
**Think**	Cognitive disruption; avoidance	Attention, memory, executive function performance	Smartphone-based cognitive tasks (e.g., N-back, MoCA digital)	Monitor cognitive impact of PTSD; identify avoidance of trauma cues; evaluate treatment response
**See**	Hypervigilance; sleep disturbance; visual attention	Sleep efficiency; nocturnal awakenings; threat bias	Actigraphy; smartphone sleep diary; eye-tracking	Detect sleep disruption; assess hypervigilance to threat; support grounding and sleep hygiene
**Feel**	Negative mood; stress reactivity; social functioning	PCL-5; ITQ; PHQ-9; GAD-7; HRV variability	Self-report apps; ecological momentary assessment (EMA); wearable HRV and GSR sensors	Continuous monitoring of distress and affect; provide real-time grounding prompts; link to brain health capital

## Moving measurement into practice

We propose that integrating the multimodal signals described in the “walk, talk, think, see, feel” framework with digital twinning could build a personalised, dynamic profile of how PTSD symptoms manifest and fluctuate across daily life^29^. This would allow for: insight into individual impact of PTSD, understanding of non-psychological factors that maintain PTSD symptoms, earlier detection of relapse risk; real-time prompts to support grounding and emotional regulation; and can highlight links between behavioural change and reduced symptomology and gains in overall brain health capital. In this way, the “walk, talk, think, see, feel” framework provides a bridge from theory to practice: situating PTSD within a whole-system model of brain health, and demonstrating how digital biomarkers and AI can help make this complexity measurable, actionable, and ultimately more person-centred. This model could also be used to support investigations into risk factors for PTSD development in the aftermath of a traumatic event.

In practice, implementation of this framework is likely to be modular rather than requiring simultaneous measurement across all domains. Data collection can be tailored to individual needs, clinical priorities, and tolerability. Advances in digital health are also enabling increasing reliance on passive data collection (e.g., activity, sleep, and physiological signals), which may reduce participant burden and improve adherence compared to active self-report approaches. Considerations such as device burden, battery life, and sustained engagement remain critical, particularly in PTSD populations, and highlight the importance of co-design approaches. Future implementations must incorporate appropriate clinical governance, including protocols for identifying and responding to acute distress, and alignment with existing digital mental health safety standards.

## What a digital twin could add

Digital twinning provides such a method that is being rapidly deployed across many disciplines. In this context, a digital twin can be conceptualised as a dynamic, data-driven representation of an individual’s brain health state, integrating multimodal behavioural, physiological, and contextual data over time. By combining signals across the “walk, talk, think, see, feel” domains, such a model may provide a personalised and evolving representation of symptom expression, enabling improved interpretation of patterns, trajectories, and potential intervention points^29^. The application of wearables to create a digital twin of physical and psychological information for a patient with PTSD would incorporate complex information in a meaningful way that could provide a unique opportunity to identify novel associations and generate hypotheses regarding underlying mechanisms^30^ and support empowered decision-making, reducing avoidance and potentially PTSD symptoms whilst on often lengthy healthcare waiting lists. There is evidence to support that providing multimodal feedback on behavioural and physiological states, analogous to, but broader than, traditional neurofeedback approaches can improve PTSD symptoms^31,32^. Establishing causality will require complementary experimental and longitudinal study designs. Clinically, feeling empowered to identify and understand PTSD symptoms, particularly with near real-time information, would allow for earlier and more personalised insight and decision-making capacity about how to respond, offering potential to interrupt cycles of distress before they create harm.

## Conclusions

Integrating a wider systems lens of the causes and symptoms of PTSD with biomarker technology and digital twins has the potential to provide people with PTSD an opportunity to understand their unique PTSD presentation, feel empowered to apply evidence-based strategies (as prompted by a digital twin) and seek support when required. While individuals will benefit from making connections between their physical health, wellbeing and social connectedness, data gathered from wide samples can also benefit the field of PTSD research, allowing modelling of complex and interconnected facets of socio-demographics, health and behaviour. Use of digital twin technology may also illuminate differentiations and similarities between other mental health conditions. Understanding the role of non-psychological factors in PTSD maintenance can identify alternate routes of support for people with PTSD which could reduce the burden on specialist psychological therapy services, as people arrive for treatment with understanding and coping strategies in place, or indeed, no longer require treatment if alternate mechanisms activate their internal recovery mechanisms. Potential unintended consequences must also be considered. Continuous monitoring and feedback may increase symptom awareness or exacerbate hypervigilance in some individuals. Implementation should therefore follow trauma-informed principles, including user control over data visibility, adjustable feedback frequency, and the ability to disengage monitoring.

Offering choice is a fundamental principle of trauma-informed practice, and utilising technology offers individual oversight of symptoms monitoring and response. This could be used alongside or independently of a practitioner too. Next steps must involve collaboration and co-design with patients and stakeholders to identify what traits and symptoms are of most importance to people living with PTSD and C-PTSD, and what level of measurement is acceptable, tolerable and useful to those who we expect will benefit most from this new approach.

## Data Availability

No datasets were generated or analysed during the current study.
